# Diversity of Conotoxin Gene Superfamilies in the Venomous Snail, *Conus victoriae*

**DOI:** 10.1371/journal.pone.0087648

**Published:** 2014-02-05

**Authors:** Samuel D. Robinson, Helena Safavi-Hemami, Lachlan D. McIntosh, Anthony W. Purcell, Raymond S. Norton, Anthony T. Papenfuss

**Affiliations:** 1 Medicinal Chemistry, Monash Institute of Pharmaceutical Sciences, Monash University, Parkville, VIC, Australia; 2 Department of Biochemistry and Molecular Biology, Bio21 Institute, University of Melbourne, Parkville, VIC, Australia; 3 Bioinformatics Division, The Walter and Eliza Hall Institute of Medical Research, Parkville, Melbourne, VIC, Australia; The City University of New York-Graduate Center, United States of America

## Abstract

Animal venoms represent a vast library of bioactive peptides and proteins with proven potential, not only as research tools but also as drug leads and therapeutics. This is illustrated clearly by marine cone snails (genus *Conus*), whose venoms consist of mixtures of hundreds of peptides (conotoxins) with a diverse array of molecular targets, including voltage- and ligand-gated ion channels, G-protein coupled receptors and neurotransmitter transporters. Several conotoxins have found applications as research tools, with some being used or developed as therapeutics. The primary objective of this study was the large-scale discovery of conotoxin sequences from the venom gland of an Australian cone snail species, *Conus victoriae*. Using cDNA library normalization, high-throughput 454 sequencing, *de novo* transcriptome assembly and annotation with BLASTX and profile hidden Markov models, we discovered over 100 unique conotoxin sequences from 20 gene superfamilies, the highest diversity of conotoxins so far reported in a single study. Many of the sequences identified are new members of known conotoxin superfamilies, some help to redefine these superfamilies and others represent altogether new classes of conotoxins. In addition, we have demonstrated an efficient combination of methods to mine an animal venom gland and generate a library of sequences encoding bioactive peptides.

## Introduction

Animal venoms represent a vast library of bioactive peptides and proteins. This is illustrated elegantly in cone snails (genus *Conus*), a group of carnivorous mollusks that exhibits a remarkable strategy for prey capture. A cone snail injects venom into its victim using a modified radula tooth, whereby components of the venom act potently and selectively at a range of molecular targets in the victim’s nervous system to achieve incapacitation [1]. Cone snail venoms are remarkably complex, containing hundreds of unique bioactive peptides termed conotoxins (or conopeptides).

Molecular targets of individual conotoxins are diverse and include a range of voltage-gated ion channels, ligand-gated ion channels, G-protein coupled receptors and neurotransmitter transporters [2]. As such, *Conus* venoms are an excellent source of pharmacological tools crucial to fundamental neuroscience research. Moreover, conotoxins have found use as therapeutics. An example is Ziconotide (Prialt®), the synthetic equivalent of ω-MVIIA from the venom of C*onus magus*, which is being used to treat chronic pain in cancer and AIDS patients [3]. Several others also show potential and are currently undergoing development for the treatment of pathologies including postoperative and neuropathic pain, epilepsy, myocardial infarction and hypertension [4].

The epithelial cells lining the duct of a cone snail’s venom gland, are rich in messenger RNAs (mRNAs) encoding conotoxins [5]. These mRNAs are translated initially as inactive precursor peptides that require post-translational processing prior to secretion from the cell as the bioactive mature peptides [6]. Conotoxin precursors exhibit a generally recognizable primary structure: a hydrophobic signal peptide (prepeptide) sequence, followed by a propeptide region and commonly a cysteine-rich mature peptide region. The signal sequence of a precursor peptide is responsible for targeting it to the cellular secretory pathway, but is removed prior to secretion of the mature peptide. Conotoxins can be classified into gene superfamilies according to this signal peptide sequence [7]. Members of a conotoxin superfamily share a high percentage of sequence identity in their signal peptide sequence but less so in their propeptide sequence, and can be highly variable in their mature peptide sequence (often with the exception of the cysteine framework) [8]. A conotoxin’s cysteine framework refers to the characteristic arrangement of cysteine residues in its primary structure and is independent of disulfide connectivity (to date, approximately 25 distinct cysteine frameworks have been described in conotoxins). While there is no interdependence between gene superfamily and biological function [7], a conotoxin’s gene superfamily (and cysteine framework) remains a useful predictor of biological function.

The primary objective of this study was the large-scale discovery of novel conotoxin sequences from the venom gland of *C. victoriae*. The focus of this study, *C. victoriae* (Reeve, L.A., 1843) is a molluscivorous species of cone snail endemic to the coastline of north-western Australia. To date, it is best known as the source of α-conotoxin Vc1.1, a conotoxin with considerable potential for development as an analgesic drug [9]. Other than Vc1.1, 23 unique conotoxin sequences from only a few gene superfamilies (A, O1, O2, T) are known from this species [10]–[13]. Here we report the discovery of over 100 unique conotoxin sequences from 20 gene superfamilies. Many of the sequences identified are new members of known superfamilies and some will help to redefine these superfamilies. Other sequences represent altogether new classes of conotoxins. This work paints a comprehensive portrait of the molecular diversity present in *Conus* venom.

## Results

### Sequencing, Assembly & Annotation

RNA was extracted from the venom gland of *C. victoriae*. A normalized cDNA library was generated and sequenced using the Roche 454 platform. Sequencing yielded (following clipping to remove 454 adapter sequences) a total of 701,536 reads (265,403,303 nucleotides (nt), minimum length: 2 nt, average length: 378 nt, median length: 419 nt, maximum length 920 nt).

Assembly with MIRA produced 40,513 contigs (from 463,701 reads longer than 30 nt) with an average length of 588 nt (median: 528 nt), a maximum of 7,406 nt and minimum of 30 nt (user-defined). A general annotation of the transcriptome using BLASTX [14], [15] revealed 7,818 contigs with significant similarity to sequences in the reference databases (UniProt/SwissProt and ConoServer [7]).

While BLASTX was used for a general annotation of the transcriptome, profile hidden Markov models (pHMMs) were used (independently of BLAST) to annotate conotoxins. pHMM models were built based on known conotoxin superfamilies (as described in methods) and used to search the *C. victoriae* venom gland transcriptome. Briefly, 2,048 contigs (26%) were identified (using pHMM searches) as conotoxin-encoding (combined total from all superfamilies). In terms of sequencing reads, of those that were assembled, 100,846 (22%) corresponded to conotoxins. A total of 113 conotoxins was identified from 20 superfamilies, which are described in detail below.

The *C. victoriae* cDNA library was subjected to normalization in an effort to enhance the diversity of transcripts observed. Normalization refers to a process by which distinct cDNAs are equalized and is useful to identify genes transcribed at a relatively low level (in a cellular transcriptome the number of mRNA copies per gene may differ by several orders of magnitude [16]). Normalization has the effect of “dampening down” highly abundant transcripts and consequently increasing the proportion of reads encoding rare transcripts [17]. We opted to utilize normalization as the goal of this study was to maximize the number of unique conotoxin transcripts identified. One consequence of normalization is that the number of sequencing reads no longer directly reflects transcript expression level. However, it is not expected to alter the rank order of gene expression, such that a highly abundant transcript will still be represented by the highest number of reads while a low abundance transcript will be represented by few. With this in mind, we investigated those contigs that were generated from the highest number of sequencing reads. Conotoxins made up the majority of high-ranking contigs (45 of the top 50 annotated contigs). The 10 contigs with highest read coverage included the four conotoxins Vc5.1, Vc1.1, Vc5.3 and T_Vc5.9 (described in detail below), as well as two contigs with significant similarity to each of the cytochrome c oxidase subunits 1 and 2 [UniProt: Q34941, P00409] and a contig with significant similarity to the human mucin-6 protein [UniProt: Q6W4X9], a secreted protein that plays an important role in the protection of epithelial tissues. Most of other high-ranking non-toxin contigs were associated with the processing and transport of secreted proteins. These included several potential chaperones of the heat shock protein family [UniProt: P08712, Q16956, Q05557, Q71U34, P19120, Q9Y3Q3, P41827], protein disulfide isomerases [UniProt: P09103, P05307] and a neuroendocrine convertase [P63240]. Two contigs with significant similarity to proteins of the transposase 5 family were present [UniProt: P35072, P03934]. Also present was a contig sharing significant sequence similarity with the angiotensin-converting enzyme (ACE) [UniProt: Q50JE5]. ACE converts angiotensin I to angiotensin II, with a resultant increase in vasoconstrictor activity. Its presence here raises the possibility of a role in envenomation.

### Conotoxin Gene Superfamilies

#### A-superfamily

**Figure 1:**
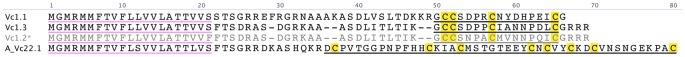
Translated *C. victoriae* A-superfamily precursor sequences. *, Vc1.2 precursor [10] shown for comparison is in grey; Cys, yellow; Predicted signal peptides are underlined in purple and the predicted mature peptides are underlined in black, while that of Vc1.2 is underlined in grey. This color scheme is used in all subsequent figures.

A pHMM was built based on the sequences of known A-superfamily conotoxins and used to search the *C. victoriae* venom gland trancriptome. This enabled the identification of a cDNA sequence encoding the peptide precursor of a novel A-superfamily conotoxin (Figure 1). This precursor shared obvious homology with other A-superfamily conotoxins, at least in its signal peptide sequence, although the sequence encoding the mature peptide is clearly novel. A_Vc22.1 is the first A-superfamily peptide to exhibit the type XXII cysteine framework (i.e. 8 cysteine residues separated by 7 loops: C-C-C-C-C-C-C-C). Several conotoxin precursor sequences with this framework have been identified in *Conus californicus* [18], although they share very little sequence similarity with A_Vc22.1, and do not belong to the A-superfamily. No conotoxin with framework XXII has been characterized to date and A_Vc22.1 offers an exciting prospect as a functionally novel conotoxin.

Other A-superfamily peptide precursor sequences identified in the venom gland transcriptome of *C. victoriae* were those of Vc1.1 [19] and Vc1.3 [10] (Figure 1). Vc1.1 is a potent analgesic in neuropathic pain models [9] and targets both the α9α10 nAChR and the γ-aminobutyric acid (GABA)_B_ receptor [20], while Vc1.3, which was identified previously in embryonic *C. victoriae*, had little effect at either the nAChRs subtypes tested or at the GABA_B_ receptor [10]. Vc1.1 is, to date, the only conotoxin from the venom of *C. victoriae* with a defined molecular target. The naming of conotoxin precursors is described in the Discussion.

#### I1-superfamily

**Figure 2:**
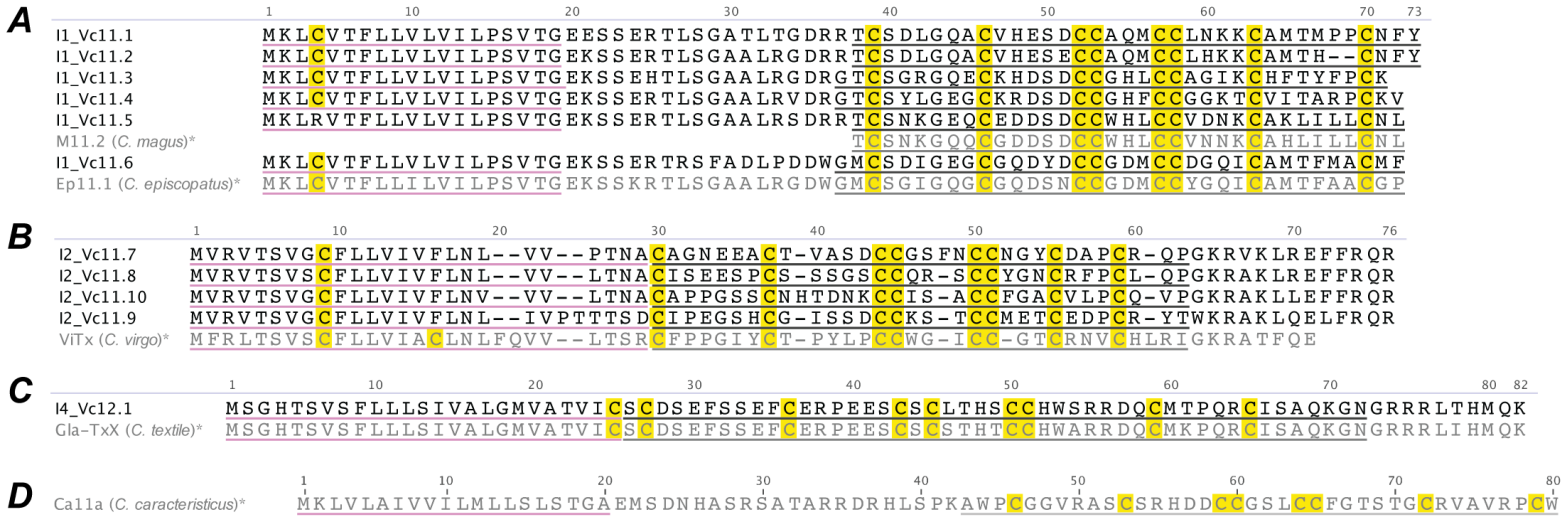
Translated *C. victoriae* I1-superfamily (A), I2-superfamily (B) and I4-superfamily (C) precursor sequences. *, M11.2 mature peptide [24], Ep11.1 [25] precursor, ViTx precursor [26], Gla-TxX precursor [77]and the I3-superfamily (D) precursor Ca11a [60]shown for comparison.

Six unique I1-superfamily conotoxins were identified in the venom gland transcriptome of *C. victoriae* (Figure 2A). I1-superfamily conotoxins characterized so far display excitatory activity [21], some through subtype-specific modulation of voltage-gated Na^+^ channels [22], [23]. The predicted mature peptide sequence of I1_Vc11.5 shares 89% identity with an I1-superfamily conotoxin from *Conus marmoreus* (M11.2) [24], while that of I1_Vc11.6 shares 82% identity with an I1-superfamily conotoxin from *Conus episcopatus* (Ep11.1) [25]. The remaining sequences I1_Vc11.1–4 do not show any notable similarity, other than their cysteine framework, to known sequences.

#### I2-superfamily

Four unique I2-superfamily conotoxins were identified (Figure 2B). They displayed the same precursor structure as those identified previously with a *C*-terminal propeptide region and a mature peptide region characterized by cysteine framework XI (C-C-CC-CC-C-C). All I2-superfamily conotoxins characterized so far (BtX, ViTx and sr11a) are K^+^ channel modulators [26]–[28]. Of the sequences identified here, there is little similarity in the mature peptide regions to known sequences. One can only speculate that, like their counterparts, these peptides would share the ability to modulate K^+^ channels, although the lack of similarity presented in their mature peptide sequences makes it is quite possible, as observed with other conotoxin superfamilies, that they display altered selectivity.

#### J-superfamily

**Figure 3:**
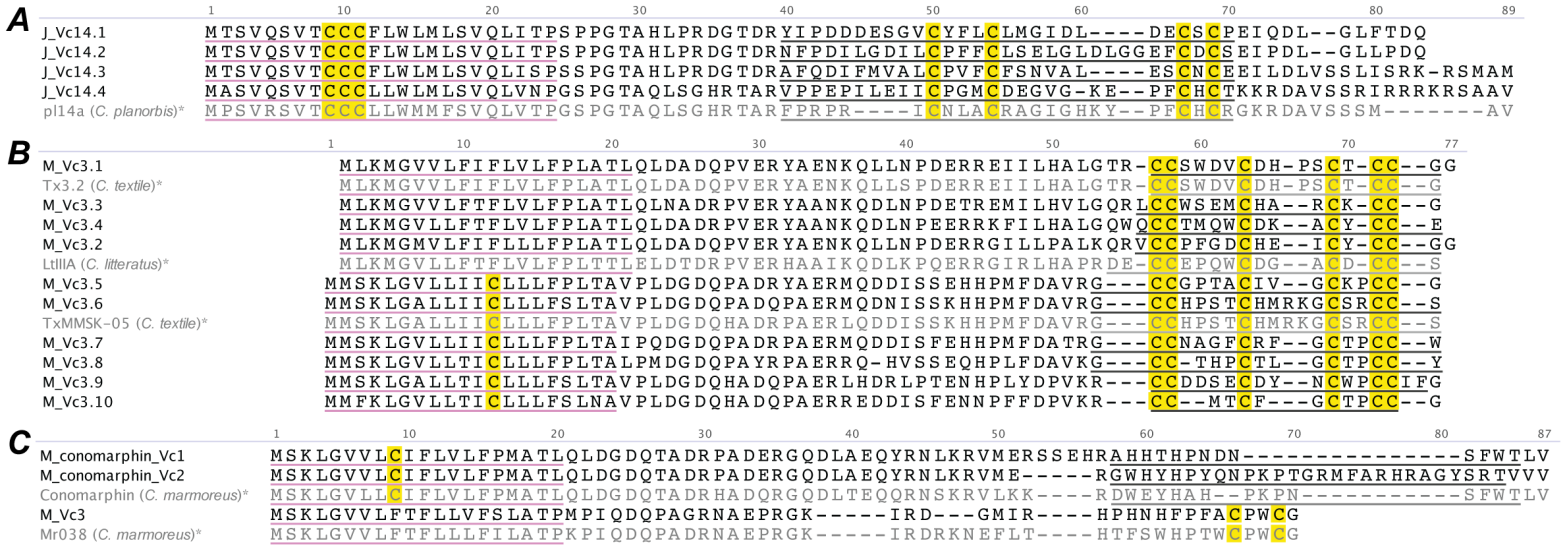
Translated *C. victoriae* J-superfamily (A), M-superfamily (B), M-conomarphin (C) precursor sequences. *, pl14a [29], Tx3.2 (tx3a) [32], TxMMSK-05 [78], LtIIIA [33], conomarphin [79], Mr038 [34] precursors shown for comparison.

Four unique J-superfamily conotoxins were identified in the venom gland transcriptome of *C. victoriae* (Figure 3A). These sequences displayed only superficial similarity to known J-superfamily sequences (specifically cysteine framework). The only J-superfamily conotoxin characterized as yet, pl14a, was observed to have a potent inhibitory affect at both nicotinic acetylcholine receptors (α3β4-neuronal, α1β1εδ-neuromuscular) and a voltage gated K^+^ channel subtype (Kv1.6) [29]. Given the low similarity between pl14a and the sequences identified here one can only speculate as to their activity. However, we note that the J-superfamily makes up a large proportion of the conotoxin mRNA transcripts observed in the venom gland of *C. victoriae*.

#### M/conomarphin-superfamily

Several conotoxin sequences from each of the M1, M2 and conomarphin subgroups of the M-superfamily were identified (Figure 3B and C). M4 and M5 conotoxins are believed to be absent from mollusc-hunting *Conus* [30], and indeed were not identified in *C. victoriae*.

Almost all of the M-superfamily sequences identified in *C. victoriae* (M_Vc3.1–2, 4–10) were very similar if not identical to previously reported M-superfamily sequences. While the M4/5 branch of conotoxins is well characterized, there are limited published data describing the M1 and M2 branches. Of the M1/M2 conotoxins tested so far, the majority elicited excitatory symptoms upon intracranial (IC) injection in mice [31], [32], while LtIIIA enhanced tetrodotoxin-sensitive Na^+^ currents in a whole-cell patch-clamp assay [33].

The M_conomarpin_Vc1 and M_conomarpin_Vc2 sequences clearly belong to the cysteine-free conomarphin class of conotoxins, although the predicted mature peptides of each differ substantially from previously identified conomarphins. M_Vc3, along with a sequence recently identified in *C. marmoreus* (Mr038) [34], presumably constitutes a new class of single disulfide-containing conotoxins.

#### O1-superfamily

The O1-superfamily of conopeptides consists of δ- (which block inactivation of voltage-gated Na^+^ channels), μ- (voltage-gated Na^+^ channel blockers), κ- (voltage-gated K^+^ channel blockers) and ω-conopeptides (voltage-gated Ca^2+^ channel blockers), all of which share a type VI/VII cysteine framework (C-C-CC-C-C).

**Figure 4:**
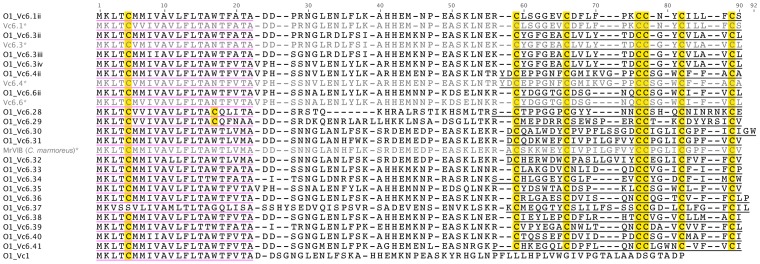
Translated *C. victoriae* O1-superfamily precursor sequences. *, Vc6.1, Vc6.3, Vc6.4, Vc6.6 [11] and MrVIB [80] shown for comparison.

Several O1-superfamily sequences have been identified previously in *C. victoriae* [11], [13]. Surprisingly, while many O1-superfamily sequences were identified here (Figure 4), none matched exactly those identified previously. Minor variants of Vc6.1, Vc6.4 and Vc6.6 were present that displayed up to three differences each in their prepropeptide regions. As there was no change in the mature peptide sequence we have denoted these sequences as variants e.g. O1_Vc6.1ii. A sequence clearly similar to Vc6.2 was also evident (with minor variation); because some of this variation occurred in the predicted mature peptide region, however, this sequence was designated as unique (O1_Vc6.41). Three unique variants of Vc6.3 were present, none of which corresponded exactly to the original Vc6.3. Again the variation occurred in the prepropeptide region and the predicted mature peptide region remained unchanged.

The remaining O1-superfamily sequences identified were completely novel, although some showed similarity to known ω-, δ-, and μ-conotoxins. Notably, the predicted mature peptide sequence of O1_Vc6.31 was 90% identical to μ-MrVIB, an O1-superfamily conotoxin from *C. marmoreus* that is an inhibitor of the Na_V_1.8 subtype of voltage-gated Na^+^ channels with analgesic properties [35].

A single cysteine-free sequence (O1_Vc1) from the O1-superamily may constitute a new class of conotoxin. Close inspection of the sequencing reads encoding this transcript (taking into account contig coverage and read quality) indicated that this unusual sequence was not simply the result of a frameshift due to sequencing error.

#### O2/contryphan-superfamily

Eleven O2 conotoxin precursors were identified previously by cDNA sequencing of the *C. victoriae* venom gland and designated Vc6.7–17 [10].

**Figure 5:**
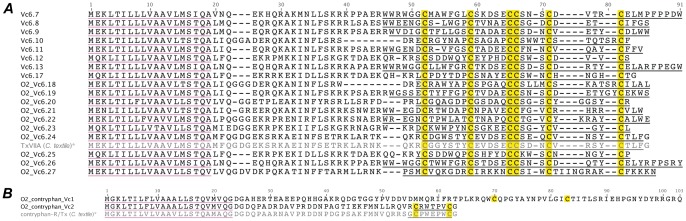
Translated *C. victoriae* O2-superfamily (A) and contryphan (B) precursor sequences. *, TxVIIA [78], [81] and contryphan-R/Tx precursors [82] shown for comparison.

A pHMM was built based on the sequences of all known O2/contryphan-superfamily conotoxins and used to search the *C. victoriae* venom gland transcriptome. 18 unique O2-superfamily (cysteine framework VI/VII) and two contryphan conotoxins were identified (Figure 5A and B). Of the 16 O2-superfamily conotoxins identified with cysteine framework VI/VII, eight had been identified previously. A minor variant of Vc6.16 was also evident, with a single difference in the predicted mature peptide region (this sequence was therefore designated O2_Vc6.25). The predicted mature peptide sequence of O2_Vc6.22 was 81% identical to TxVIIA, a modulator of molluscan pacemaker channels (γ-conotoxin) [36].

Contryphans are short single disulfide-containing conotoxins that display a diversity of function but could generally be described as Ca^2+^ channel modulators [37], [38]. Both of the contryphans identified share obvious homology, at least in their signal peptide sequence, to other contryphans, although the sequences encoding the mature peptides are clearly novel. Contryphan_Vc1 is the first contryphan peptide identified that exhibits an intercystine loop length other than five residues. Indeed, this peptide is remarkably different in its entire primary structure from any conotoxin previously characterized.

All contryphans identified so far have either Pro/Hyp followed by D-Trp or Val followed by D-Leu at positions one and two of the intercystine loop. Hyp (or Pro) at position 1 of the disulfide loop appears to be necessary for slow conformational interconversion observed in these peptides [39]. The precursor cDNA sequence of contryphan_Vc2 indicates that this peptide has a Trp at position two (presumably D-Trp [40]) but is unique among contryphans in that it exhibits the positively-charged amino acid Arg at position one. Its sequence also differs from other known contryphans at positions 3 and 5 (Thr and Val, respectively). Further characterization of this peptide is likely to offer important information on the structure-activity relationship of contryphans.

Other than its propeptide sequence and single pair of cysteines, contryphan_Vc1 shares no obvious sequence similarity to contryphan_Vc2, or indeed any other contryphans.

#### O3-superfamily

**Figure 6:**
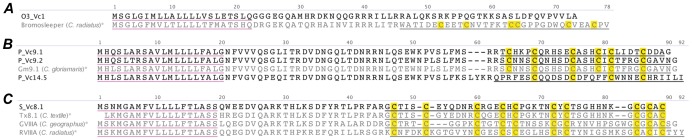
Translated *C. victoriae* O3-superfamily (A), P-superfamily (B) and S-superfamily (C) precursor sequences. *, Bromosleeper peptide (GenBank: GQ981406.1) [41], Gm9.1 (GmIXA) [43], Tx8.1 [46], GVIIIA [44] and RVIIIA [45] precursors shown for comparison.

One O3 superfamily precursor was identified in *C. victoriae* (Figure 6A). The signal peptide sequence indicated that this sequence was related to the O3-superfamily, although the pro- and mature peptide regions differed markedly from known O3-superfamily sequences, most notably in that it was devoid of cysteines, in contrast to all O3-superfamily conotoxins identified to date, which are cysteine-rich with framework VI/VII, e.g. the bromosleeper peptide [41].

#### P-superfamily

Three P-superfamily precursor sequences, P_Vc9.1, P_Vc9.2 and P_Vc14.5, were identified in the venom gland transcriptome of *C. victoriae* (Figure 6B). While P_Vc9.1 and P_Vc9.2 display the type IX cysteine framework (C-C-C-C-C-C) consistent with previously identified P-superfamily conotoxins [42], [43], P_Vc14.5 displays a type XIV cysteine framework (C-C-C-C). Alignment of this sequence with the two type IX peptides indicates that the equivalent II–V and III–VI cysteine pairs are still present but the I–IV cysteine pair is absent.

The predicted mature peptide sequence of P_Vc9.2 is 96% identical to GmIXA, a conotoxin from the venom of *Conus gloriamaris* that induces hyperactivity and spasticity in mice following IC injection [43]. Like the J-superfamily, the relatively uncharacterized P-superfamily appears to constitute a large proportion of conotoxin mRNA transcripts in the venom gland of *C. victoriae*.

#### S-superfamily

The two S-superfamily conotoxins to have undergone pharmacological characterization displayed different activity: GVIIIA competitively inhibited the 5-HT_3_ serotonin receptor [44], while αS-RVIIIA inhibited nAChRs [45]. A single S-superfamily precursor sequence, S_Vc8.1 was identified in the venom gland transcriptome of *C. victoriae* (Figure 6C). The peptide shared the same cysteine framework as previously identified S-superfamily conotoxins. The predicted mature peptide sequence of S_Vc8.1 shares 93% identity with that of tx8.1 from *Conus textile* [46].

#### T-superfamily

**Figure 7:**
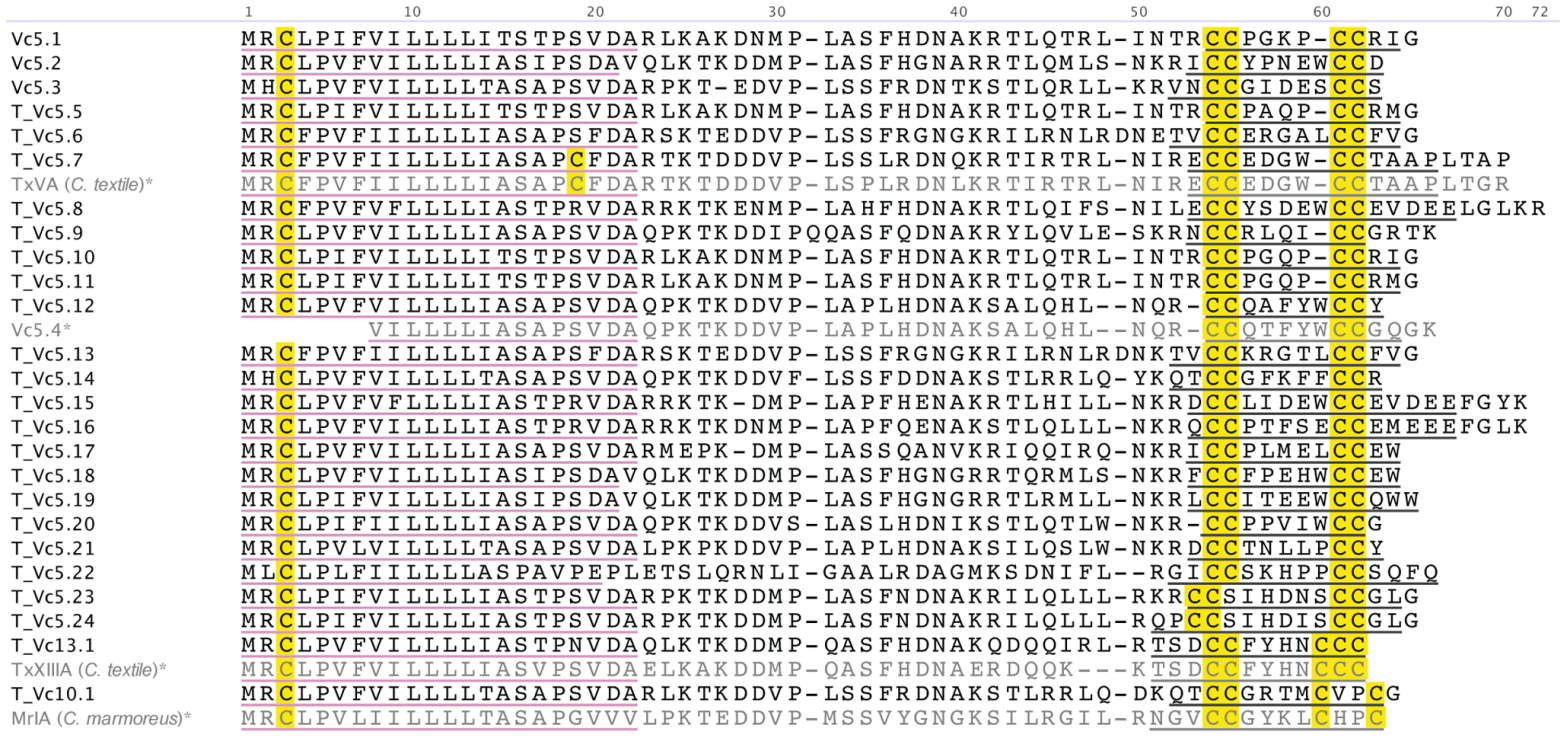
Translated *C. victoriae* T-superfamily precursor sequences. *, Vc5.4 [11], TxVA [78], TxXIIIA [47], and χ-MrIA [83] precursors shown for comparison.

The precursor sequences of 27 unique T-superfamily conotoxins were identified (Figure 7), making it not only the most abundant superfamily in *C. victoriae*, but also the most diverse. Three different cysteine frameworks (V, X and XIII) were identified.

Three of the 27 sequences had been identified previously in *C. victoriae* venom duct mRNA, while the predicted mature peptide sequences of two others, T_Vc5.7 and T_Vc13.1, had been identified previously in the venom of *C. textile.* The predicted mature peptide sequence of T_Vc13.1 was identical to TxXIIIA, a unique T-superfamily conotoxin identified in *C. textile* [47]. This peptide is similar to the Type V framework (CC-CC) conotoxins, but contains an extra Cys (CC-CCC), and is found in the venom as a homodimer. The predicted mature peptide sequence of T_Vc5.7 was identical to TxVA, one of the most highly modified conotoxins, with γ-carboxyglutamate, hydroxyproline, bromotryptophan and glycosylation [48], [49]. This conotoxin induces hyperactivity and spasticity in mice following IC injection, and may target a pre-synaptic Ca^2+^ channel or GPCR. One T-superfamily sequence identified in *C. victoriae* venom gland mRNA in a previous study [11], Vc5.4 (Vc5c), was not identified here, although a very similar sequence (T_Vc5.12) was present. T_Vc10.1 shares obvious homology with known χ-conotoxins (inhibitors of the noradrenaline transporter), in both its T-superfamily signal peptide and mature peptide sequences.

Despite evidence that the T-superfamily is abundant, not only in *C. victoriae* but in other species of *Conus* as well, remarkably little is known about this group of conotoxins [50].

#### Conantokins (B-superfamily)

**Figure 8:**
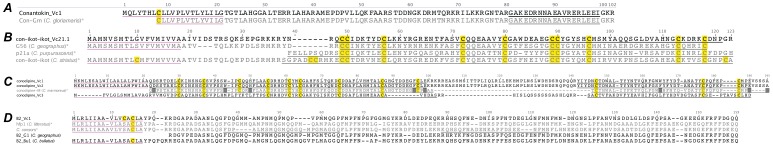
Translated *C. victoriae* conantokin (A), con-ikot-ikot (B), conodipine (C) and B2-superfamily (D) precursor sequences. *, Con-Gm [51], G56 [5], con-ikot-ikot [52], p21a [53], Conodipine-M [54] and B2-superfamily sequences from *C. literratus* [57] and *C. consors* [56] are shown for comparison. The conodipine catalytic His-Asp dyad is boxed in red.

A pHMM was constructed based on the sequences of known conantokin precursors and was used to search the *C. victoriae* venom gland transcriptome. This search yielded a single conantokin transcript (Figure 8A). An almost identical sequence (only three changes in the predicted prepropeptide region) has been reported in another molluscivorous species, *C*. *gloriamaris* (Con-Gm) [51]. The mature form of Con-Gm is reportedly 19 amino acids in length, with residues Glu4, Glu10 and Glu14 being modified to γ-carboxyglutamate and the *C*-terminus being amidated.

#### Con-ikot-ikots

The original con-ikot-ikot was identified and characterized from the venom of the *Conus striatus* [52]. Uniquely among conotoxins, it displayed an effect on α-amino-3-hydroxy-5-methyl-4-isoxazolepropionic acid (AMPA) receptors, inhibiting channel desensitization. Con-ikot-ikot is a relatively large conotoxin with 13 cysteine residues, where the active form is a dimer of covalent dimers.

A recently discovered conotoxin isolated from the venom of *Conus purpurascens*, p21a, showed 48% homology with con-ikot-ikot [53]. p21a defined a new 10-cysteine, 7-loop framework (XXI), a similar cysteine arrangement to con-ikot-ikot. Unlike con-ikot-ikot, however, this conotoxin has been proposed to form a non-covalent dimer. Multiple con-ikot-ikot precursor sequences were also recently identified in the venom gland transcriptome of *Conus geographus* [5], three of which shared framework XXI with p21a, and two displayed the original con-ikot-ikot framework.

Here we show that con-ikot-ikots are not limited to the fish-hunting species described above. A con-ikot-ikot precursor sequence was identified in *C. victoriae* (Figure 8B). This sequence displayed the same cysteine framework (XXI) as p21a.

#### Conodipines

Secretory phospholipase-A_2_s (sPLA_2_s) have been reported in a wide variety of animal venoms, as well as mammalian tissues and bacteria. They catalyze the hydrolysis of the ester bond at the *sn-*2 position of 1,2-diacyl-*sn*-phosphoglycerides. In addition to enzymatic activity some of these venom PLA_2_s display potent neurotoxicity.

Conodipine-M, a 13.6 kDa component of the venom of *C. magus* [54], was until now the only phospholipase characterized from *Conus* venom, although various conodipine isoforms are reportedly present in the venom gland transcriptome of *Conus consors* [55]. Its sequence was partially characterized and differed from most other conotoxins in that it was present as a heterodimer of two polypeptide chains, an α- and a β-chain. Conodipine-M displayed sPLA_2_ activity and like other sPLA_2_s, required Ca^2+^ as a cofactor [54]. Its sequence, while retaining key catalytic motifs present in other sPLA_2_s, shared little sequence identity with other sPLA_2_s and therefore defined a new group (IX) of enzymes.

Here we show that conodipines, like other sPLA_2_s, are encoded by a single precursor consisting of a signal peptide sequence followed by the α-chain, a propeptide linker and finally the β-chain (Figure 8C).

Two of the precursors identified display remarkable similarity in their predicted mature peptide region to conodipine-M, including their cysteine framework and catalytic His-Asp dyad. The remaining sequence retains the general precursor structure of conodipine_Vc1 and 2 and the predicted catalytic dyad, but displays not only a unique signal peptide sequence but also a unique cysteine framework. Given its unique signal peptide sequence, this conotoxin could be considered the first member of a new superfamily.

### New or Recently Identified Conotoxin Superfamilies

#### B2-superfamily

In a previous study, several linear peptides identified in the venom proteome of *C. consors* were matched to a sequence in the transcriptome that did not correspond to a known conotoxin superfamily [56]. Interestingly, a similar sequence (UniProt Q2HZ30) had been identified at high frequency in a *Conus litteratus* venom gland cDNA library [57]. Although the function of the peptide products of these sequences remains unknown, the authors proposed that these sequences may constitute an as yet undescribed conotoxin superfamily. Recently, a similar sequence was identified in the venom gland transcriptome of *C. marmoreus* and subsequently designated as the B2-superfamily [34].

Based on alignment of two known B2-superfamily precursor sequences from *C. litteratus* and *C. consors*, a pHMM was built and used to search the transcriptome of *C. victoriae*, as well as the transcriptomes of *Conus bullatus* [58] and *C. geographus* [5]. Each species yielded a single B2-superfamily precursor sequence displaying remarkable similarity to those from *C. consors* and *C. litteratus* (Figure 8D). As observed in *C. litteratus*, B2_Vc1 is observed at high frequency in the venom gland transcriptome of *C. victoriae*.

#### E- and F-superfamilies

The E- and F-superfamilies of conotoxins were recently described from the venom gland transcriptome of *C. marmoreus* [34], with each superfamily consisting at present of a single sequence. The peptide product of the only E-superfamily precursor so far identified (Mr104), is 26 amino acids in length, with four cysteines (two disulfide bonds) and a bromotryptophan. A peptide product was also identified for the F-superfamily precursor (Mr105). This short linear peptide was derived from the predicted propeptide sequence.

**Figure 9:**
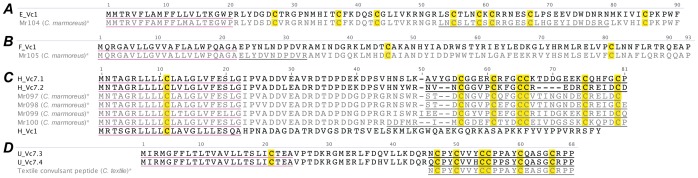
Translated *C. victoriae* E-superfamily (A), F-superfamily (B), H-superfamily (C) and U-superfamily (D) precursor sequences. *, Mr104 [34], Mr105 [34], other H-superfamily precursors (Mr097, Mr098, Mr099 and Mr100) [34]and the textile convulsant peptide [63] are shown for comparison.

pHMMs were constructed based on each of the known precursor sequences and used to search the *C. victoriae* venom gland transcriptomes for E- and F- superfamily conotoxins. As with *C. marmoreus*, single transcripts for each of the E- and F- superfamilies were present in *C. victoriae* (Figure 9A and B), which showed remarkable similarity to those present in *C. marmoreus* (Mr104 and Mr105). The venom gland transcriptomes of *C. bullatus* and *C. geographus* were also searched, using the same method, for E- and F- superfamily conotoxins, although none were identified in these species.

#### H-superfamily

The precursor sequences of several novel conotoxins clearly belonged to the recently discovered H-superfamily of conotoxins from *C. marmoreus* [34] (Figure 9C). Superficially, the cysteine pattern observed in H_Vc7.1 and H_Vc7.2 is identical to that of the O1- and O2-superfamilies. However, closer comparison reveals that there is little similarity in either the intercysteine loop composition or length [59]. The hitherto uncharacterized H-superfamily constitutes a large proportion of conotoxin mRNA transcripts in the venom gland of *C. victoriae*.

A single H-superfamily sequence encoding a cysteine-free predicted mature peptide region was also encountered (H_Vc1), indicating that, like other superfamilies, the H-superfamily is not limited to a single cysteine framework. This unusual sequence probably constitutes a new class of conotoxin. As described above for O1_Vc1, a close inspection of the sequencing reads was performed to confirm that this unusual sequence was not simply the result of a frameshift due to sequencing error.

#### I4-superfamily

A recently described third I-superfamily (I3) [60] (Figure 2D), was searched for but not identified in the venom gland of *C. victoriae*. However, during the process of designing and building each I-superfamily pHMM, it became apparent that a fourth, unrecognized, superfamily of conotoxins was presently grouped into the I2-superfamily. These sequences included Gla-TxX from *C. textile* [61] and Gla-MrII from *C. marmoreus* [61], the mature peptides of which are 47 and 50 residues, respectively, each with 5 γ-carboxyglutamate modifications. Not only do these conotoxins have a clearly distinct signal peptide sequence but they also exhibit a distinct cysteine framework, XII (C-C-C-C-CC-C-C), compared to other I-superfamily conotoxins [61]. This disparity has been noted previously [62], and it was proposed that this group of peptides be redefined as ‘E-conotoxins’. As an E-superfamily has since been described, and given the similarity of these conotoxins to other I-superfamilies, we propose a new I4-superfamily, which would include, among others, Gla-TxX, GlaMrII and the sequence identified in *C. victoriae* described below.

Construction of a pHMM based on these sequences enabled the identification of a single I4-superfamily member in the venom gland transcriptome of *C. victoriae* (Figure 2C). The predicted mature peptide sequence of this peptide was 92% identical to Gla-TxX. I4_Vc12.1 shares the glutamate sites of Gla-TxX, so is probably present in the venom in a similarly modified form.

#### U-superfamily

Annotation of the *C. victoriae* venom gland transcriptome with BLAST+, identified two sequences with homology to the “textile convulsant peptide” isolated two decades ago from the venom of *C. textile* [63] (Figure 9D). The textile convulsant peptide, on IC injection in mice, induces symptoms characterized by “sudden jumping activity followed by convulsions, stretching of limbs and jerking behavior”. The authors noted that this peptide was unique and predicted that it belonged to a new undefined class of conotoxins. In this study we have identified the precursor sequence of two similar conotoxins from *C. victoriae,* and shown that they are indeed members of a previously undefined conotoxin superfamily, which we have designated the U-superfamily.

Although the pre- and propeptide sequences clearly differ from known conotoxin superfamilies, the U-superfamily peptides share the cysteine framework (VI/VII) of most members of the O1-, O2- and O3-superfamilies, as well as the H-superfamily. However, on comparison with these superfamilies it is apparent that there is little similarity either in the intercysteine loop composition or length [59]. For instance, loop 1 of the U-superfamily peptides is relatively short at two residues, compared with six in the O-superfamily conotoxins.

Discovery of the signal peptide sequence for this superfamily should allow the rapid identification of U-superfamily conopeptides in other *Conus* species. With this in mind, we searched transcriptome databases of both *C. geographus* [5] and *C. bullatus* [58]. This search did not yield any hits, suggesting that this superfamily is not present (at least in high-abundance) in the fish-hunting cone snails *C. geographus* and *C. bullatus.*

Given the sequence similarity in the mature peptide sequences of U_Vc7.3 and 7.4 to the textile convulsant peptide, it is likely that they share similar biological activity. Despite its potent biological activity, the molecular target of the textile convulsant peptide has not been identified.

#### Augerpeptide Hhe53

While the venoms of *Conus* species have been rigorously investigated, those of other venomous snails remain largely unstudied. A recent investigation of the venomous Auger snail *Hastula hectica* revealed several venom peptides (termed augerpeptides) similar to those found in *Conus* venom as well as various venom gland transcripts apparently encoding other venom peptides [64]. Of the few augerpeptides identified, no overlap with conotoxins has so far been reported.

**Figure 10:**
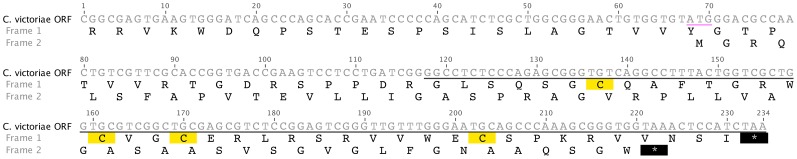
*C. victoriae* Hhe53-like open reading frame displaying translation of forward frames 1 and 2. Possible initiator codon in frame 2 is underlined in purple and the sequence encoding the predicted mature peptide in frame 1 is underlined in black.

Annotation of the venom gland transcriptome of *C. victoriae* with BLAST facilitated the identification of a contig with significant similarity to the augerpeptide hhe53 (Figure 10), a 38-residue peptide with two disulfide bonds, predicted from cDNA sequencing of the venom gland of the Auger snail *Hastula hectica*. In fact, the reported amino acid sequence of hhe53 was 100% identical to a translated region in an open-reading frame of the *C. victoriae* transcript. Investigation of the *C. victoriae* transcript revealed a stop codon in the expected position following the predicted mature peptide region as well as an Arg residue immediately 5′ to the predicted mature peptide region, indicating a possible cleavage site. However, neither an obvious signal peptide nor translation initiation codon was evident in the same open-reading frame (frame 1). The assembled contig did not suffer from low coverage (69 reads), implying that the absence of a signal peptide was not the result of a simple frameshift caused by sequencing error. We did observe, however, the presence of a possible partial signal peptide with an initiation codon in a separate reading frame (frame 2), immediately 5′ to the predicted mature peptide. We have observed elsewhere in other conotoxin sequences a naturally occurring missing propeptide region (presumably a separate exon) causing the obvious signal peptide and mature peptide regions to appear in different reading frames when translated (unpublished observation). Without a reference precursor sequence, however, it is not possible to confirm that this is the explanation for the result observed here. It remains a possibility that this presumably inactive sequence results from a polymorphism in the individual from which the mRNA was collected and that in other individuals this transcript may encode the functional peptide. The functional relevance of this sequence in *C. victoriae* therefore remains open to speculation, but the observation of an overlapping sequence in venom gland transcripts between *H. hectica* and *C. victoriae* does seem a striking coincidence.

#### Summary

**Figure 11:**
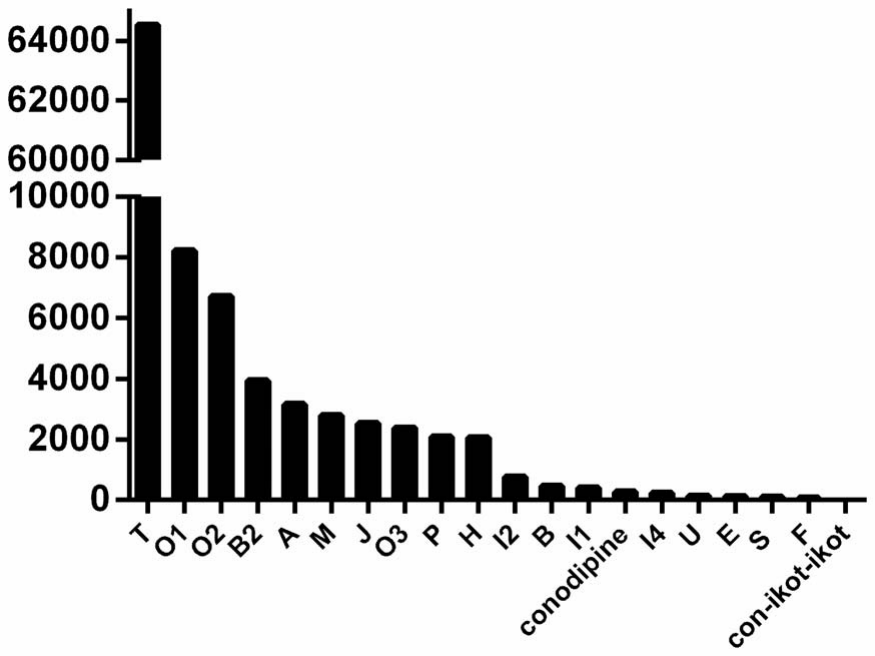
Relative abundance of conotoxin superfamilies (total reads assembled for conotoxin precursors of each superfamily). High abundance reads may be under-represented as a result of cDNA library normalization.

To give a general indication of the relative expression levels of each conotoxin superfamily in the venom gland of *C. victoriae*, reads encoding each conotoxin superfamily are presented in Figure 11. It is important to keep in mind that, owing to normalization, transcripts of high abundance may be under-represented and this chart should only be used as a general indicator.

Known superfamilies searched for, but not identified in the venom gland transcriptome of *C. victoriae* included the C, D, G, I3, K, L, N, V, Y and conopressin superfamilies. Most of these superfamilies are described from a single species or narrow range of species and it is therefore not surprising that they were not identified here in *C. victoriae*. One exception is the conopressin superfamily, identified in a number of species including the closely related *C. textile*, but not identified here.

## Discussion

The traditional approach for venom peptide identification has been assay-directed fractionation, followed by isolation and peptide sequencing. This approach is labour-intensive and requires a large amount of venom, which is not always available. The use of targeted PCR amplification of venom duct cDNA increased the speed at which venom peptides could be identified and also reduced the amount of starting material required. Similarly, large-scale cloning of cDNA libraries and Sanger sequencing has also been performed and has successfully generated a large number of novel peptide sequences [57], [65], but is relatively expensive. The recent advent of high-throughput ‘next generation’ sequencing technologies has facilitated larger, more rapid and cost-effective identification of novel venom peptides and proteins through the sequencing of venom gland transcriptomes. The potential of this approach has been recognized and applied recently to the venom gland transcriptomes of several species of *Conus* [5], [55], [58], [66]. Of the next generation sequencing platforms available, our use of 454 sequencing technology was motivated by the current superior read length generated compared to other technologies.

One trade-off, however, with this technology is the higher error rate in homopolymer runs (compared with other sequencing platforms). Such errors can result in insertions or deletions, which can introduce frameshifts or amino acid changes in the resulting sequences. For this reason reporting of 454 reads prior to assembly is risky. Higher sequence coverage provided by the assembly process works to reduce sequencing errors, producing more reliable sequences and reducing the likelihood of reporting minor variants and unusual sequences that are simply the result of sequencing error. *De novo* transcriptome assembly, however, can be a challenging task. In the assembly of the *C. victoriae* venom gland transcriptome there was evidence, particularly for the more abundant conotoxin superfamilies, that multiple contigs encoding the same transcript were generated by the assembler. In some cases this was caused by a substitution error, while others were the result of frameshifts (usually in regions of low coverage). This was also reported for the assembly of the *C. geographus* venom gland transcriptome [5]. Clustering of contigs could potentially reduce this problem, but we deemed that it was not appropriate here. A high frequency of minor variations occurs naturally in the genes encoding conotoxins (and indeed venom peptides in general) and the process of clustering is likely to mask any naturally occurring minor variations. Indeed, even without clustering, some contigs in this study were the product of two clearly unique minor variants that had been clustered by the assembler. It was necessary to perform a thorough manual examination of the contigs corresponding to each precursor sequence presented here. This was especially important for some of the minor variants and more unusual reported sequences to ensure that these were not the result of sequencing error. Researchers employing the methods described herein need to be aware of the complications associated with read error and transcriptome assembly and therefore be rigorous in their examination of, and conservative in their reporting of, unusual sequences or minor sequence variants.

Recently, it was demonstrated that pHMMs can be used to classify conotoxins and proposed that the use of pHMMs was a highly suitable approach for identifying conotoxin sequences in large datasets (e.g. transcriptomes) [67]. Here we employed pHMM searches for a more detailed investigation of the conotoxin gene superfamilies present in the venom gland transcriptome of *C. victoriae* and describe the highest diversity of conotoxins so far reported in a single study. While a number of variables could potentially contribute to this result, a comparison with a recent study performed in a similar manner but with a non-normalized cDNA library [5] suggests that our cDNA library normalization has played a major part. Hu et al., [5] investigated the venom gland transcriptome of *C. geographus*, reporting the identification of 63 unique conotoxin sequences from a dataset of 791,971 sequencing reads. From a similar dataset, in terms of total read number and average length, we report almost twice as many unique conotoxin sequences. Conotoxin sequences dominated the *C. geographus* dataset, constituting 88% of the total sequencing reads with over 250,000 of these reads encoding just three conotoxins. In our study, only 22% of the total sequencing reads encoded conotoxins, with the most abundant conotoxin, Vc5.1, comprising only 3,405 sequencing reads. In sacrificing coverage of some of our more abundant conotoxins we improved our ability to identify rarer conotoxins. Indeed, several conotoxin contigs were assembled from as few as two reads, and without a normalized cDNA library these would not have been identified. Thus, cDNA library normalization appears to be an effective strategy to maximize the identification of unique venom components.

Most of the conotoxins identified here display little amino acid sequence similarity to conotoxins with a defined molecular target. Moreover, several sequences define new classes of conotoxins and seem likely to display novel activity profiles. While each of the conotoxin precursor sequences described here is unique, several appear to encode mature peptides that are similar, if not identical, to known conotoxins (Table 1). Even subtle differences, however, in a conotoxin’s primary structure can have a dramatic effect on its function, and in most cases this is likely to be reflected in different functionality (possibly subtype selectivity or even molecular target. There seems little doubt that this library of conotoxin sequences holds a diversity of as yet undescribed functions.

**Table 1:** Functional diversity encoded in the venom gland transcriptome of *C. victoriae*.

Superfamily	Cysteineframework	# identified in*C. victoriae*	Associated activities	Reference
A	I	2	nAChRs inhibitors, GABA_B_ receptor agonists, α1-adrenoceptor inhibitor	[20], [84], [85]
	XXII	1	N.D.	
Conantokin (B)	Cysteine-free	1	NMDA receptor inhibitors	[92]
B2	Cysteine-free	1	N.D.	
E	N.D.	1	N.D.	
F	N.D.	1	N.D.	
H	VI/VII	2	N.D.	
	Cysteine-free	1	N.D.	
I1	XI	6	Voltage-gated Na^+^ channel agonists	[23]
I2	XI	4	K^+^ channel modulators	[26], [28]
I4	XII	1	N.D.	
J	XIV	4	Neuronal and neuromuscular nAChR inhibitor and voltage gatedK^+^ channel inhibitor	[29]
M1 (M)	III	4	Excitatory symptoms in mice (IC), voltage-gatedNa^+^ channel agonist	[31], [33]
M2 (M)	III	6	Excitatory symptoms in mice (IC)	[32]
Conomarphin (M)	Cysteine-free	2	N.D.	
(M)	Single disulfide	1	N.D.	
O1	VI/VII	20	Voltage-gated Na^+^ channel agonists, voltage-gated K^+^ channel blockers, voltage-gated Na^+^ channel blockers or voltage-gatedCa^2+^ channel blockers	[86]–[89]
	Cysteine-free	1	N.D.	
O2	VI/VII	18	Neuronal pacemaker modulators	[36]
Contryphan (O2)	Single disulfide	2	Ca^2+^ channel modulators	[37], [38], [90]
O3	Cysteine-free	1	N.D.	
P	IX	2	Hyperactivity and spasticity in mice (IC)	[43]
	XIV	1	N.D.	
S	VIII	1	5-HT_3_ receptor inhibitor, nAChR inhibitor	[44], [45]
T	V	24	Voltage-gated Na^+^ channel inhibitor, presynaptic Ca^2+^ channel inhibitor (or GPCR modulator), sst3 GPCR antagonist	[48], [50], [91]
	XIII	1	N.D.	
	X	1	Noradrenaline transporter inhibitors	[85]
U	VI/VII	2	convulsions, stretching of limbs and jerking behavior in mice (IC)	[63]
Con-ikot-ikot	XXI	1	AMPA receptor modulator	[52]
Conodipine		3	Phospholipase-A_2_	[54]

Each conotoxin superfamily is divided into groups according to cysteine framework, with the number identified in *C. victoriae* and a summary of biological activity associated with each group indicated.

AMPA, α-amino-3-hydroxy-5-methyl-4-isoxazolepropionic acid; GABA, γ-aminobutyric acid; GPCR, G protein-coupled receptor; IC, intracranial injection; nAChR, nicotinic acetylcholine receptor; N.D., not determined; NMDA, N-Methyl-D-aspartate; sst, somatostatin.

The naming of conotoxin precursors identified in this study was undertaken according to the conventional conotoxin nomenclature (where species is represented by one or two letters, cysteine framework by an Arabic numeral and, following a decimal, order of discovery by a second numeral) [49], with slight modifications. For previously identified conotoxin precursors the names were not altered in any way. For novel sequences we have chosen to include the superfamily as a prefix. cDNA sequencing is now the primary method for conotoxin identification, and without information on a conotoxin’s function (or even cysteine framework) the gene superfamily is becoming increasingly important for conotoxin classification. Moreover, we have made no distinction between ‘cysteine-poor’ and ‘cysteine-rich’ sequences, as this division is now considered to be largely redundant [68]. In the O1-superfamily several precursors were identified that differed in their prepropeptide but not in their mature predicted peptide regions, such that there would presumably be no difference in the peptide products of these precursors. These sequences were given the same name but a small roman numeral was added as a suffix to denote the minor variations. We suggest that the slight modifications applied here to the conventional conotoxin naming scheme should assist in the naming of new sequences identified by transcriptomic studies.

Two of the conotoxins identified here (A_Vc22.1 and P_Vc14.5) displayed cysteine frameworks not previously associated with their particular superfamily. In the case of P_Vc14.5, comparison with the primary structures of framework IX P-superfamily conotoxins suggests that this change may only be subtle. However A_Vc22.1 is not at all similar to other A-superfamily conotoxins and could therefore be expected to display a unique activity profile. Cysteine-poor conotoxins were identified in several of the traditionally cysteine-rich superfamilies (M, O1, O2, O3, and H). Other than the conomarphins and contryphans, these sequences probably represent new conotoxin classes. A con-ikot-ikot conotoxin, previously limited to piscivorous species of *Conus*, was identified here in *C. victoriae*. Additionally, a conantokin sequence was identified, providing more evidence that this superfamily is also not limited to piscivorous species of *Conus*.

Several of the relatively uncharacterized conotoxin superfamilies were observed at high abundance in the venom gland transcriptome of *C. victoriae* (H, J, P and B2). This suggests that they are key components of the venom repertoire of this species and thus warrant further investigation of their functional properties.

The goal of future studies utilizing the information presented here will be the functional characterization of the peptide products of new conotoxin sequences. The first step will be to determine the mature peptide(s) corresponding to each precursor sequence. While many mature peptide sequences and post-translational modifications can be predicted directly from a precursor sequence, some will require a more thorough examination of the venom of *C. victoriae* by tandem mass spectrometry (MS/MS) matching. To this end, the library generated here can be used as a query database for MS/MS matching against the venom of *C. victoriae*, as demonstrated recently in other *Conus* species [34], [56]. MS/MS matching will confirm mature peptide sequences and the presence of post-translational modifications. The prediction of disulfide connectivity from conotoxin precursor sequences is notoriously difficult [69], [70], and in most cases requires experimental determination. The improvement of methods for the rapid and efficient determination of a peptide’s (or protein’s) disulfide connectivity remains an active area of research [71].

## Conclusions

Given the history of the small number of conotoxins so far characterized, we predict that components discovered in this work have the potential to become valuable research tools, if not drug leads or therapeutics. This study illustrates the arsenal of molecular weapons present in the venom gland of a single species of cone snail. Furthermore, it highlights the wonderful molecular resource that is animal venom.

## Materials and Methods

### Specimen Collection and RNA Extraction

Specimens of *C. victoriae* were collected from Broome, Western Australia. Whole venom glands of live specimens were dissected, snap-frozen in liquid nitrogen and stored at -80°C. Frozen venom glands were pulverized and homogenized using an MM 400 mixer mill (Retsch). Total RNA was extracted with Trizol (Invitrogen, Life Technologies). Total RNA integrity, quantity and purity were determined by capillary electrophoresis using a Bioanalyzer 2100 with the RNA 6000 Nano assay kit (Agilent Technologies).

### cDNA Library Preparation and Sequencing

cDNA library preparation, normalization and sequencing were performed by Eurofins, MWG Operon (Budendorf, GER). From the total RNA sample, poly(A)+ RNA was isolated and used for cDNA synthesis. An N6 randomized primer was used for first strand cDNA synthesis. 454 adapters A and B were then ligated to the 5′ and 3′ ends of the cDNA, respectively. The cDNA was finally amplified by PCR (11 cycles).

Normalization was carried out by one cycle of denaturation and re-association of the cDNA. Re-associated double-stranded cDNA was separated from the remaining single stranded-cDNA (normalized cDNA) by passing the mixture over a hydroxylapatite column. After hydroxylapatite chromatography, the single-stranded cDNA was PCR amplified (8 cycles). cDNA in the size range of 500–1100 nt was eluted from a preparative agarose gel for sequencing. 454 sequencing was performed using GS FLX+ chemistry.

### Assembly

During the assembly process, single reads are aligned with each other to form contigs (contiguous consensus sequences). All reads were initially trimmed to remove primer and barcode sequences. Reads were then cleaned using prinseq-lite-0.17.1 [72]. *De novo* transcriptome assembly was performed using the following settings in MIRA3 [73]: mira -job = denovo,est,accurate,454 454_SETTINGS -CO:fnicpst COMMON_SETTINGS -GE:not = 6 -AS:nop = 4:sep = 1 -CL:ascdc = 1 454_SETTINGS -LR:lsd = 1:ft = fastq -AS:mrl = 30 -CL:cpat = 1. Based on a recent comparison of 454 assembly methods, MIRA and newbler were identified as the leading *de novo* transcriptome assemblers [74], with MIRA being more conservative about merging reads into contigs. To avoid over-assembly in the first instance, in order to identify as many alleles and paralogues as possible, we selected MIRA as our assembler. A database of open reading frames longer than 40 amino acids was generated from the transcriptome assembly. This database was used for subsequent pHMM searches.

### Transcriptome Annotation with BLAST+

For a general annotation of the transcriptome we utilized BLAST+ (version 2.2.27+) [14], [15]. Reference databases were constructed from the current UniProt/swissprot database (release 2012_09) and the non-redundant ConoServer database [7]. Each contig from the assembled transcriptome was aligned to the two databases using BLASTX (E-value cutoff: 10^−3^) and the combined best hit used. Ties were resolved by taking the ConoServer hit preferentially.

### Conotoxin Gene Superfamily Annotation with pHMMs

All conotoxin sequences available from ConoServer were downloaded and grouped according to superfamily (classification provided by ConoServer). Any identical sequences were removed. Full-length precursor sequences were used where available, but for superfamilies with less sequence information all available sequences were used.

Using the hmmbuild tool from the HMMER 3.0 package a single pHMM was built for each superfamily. The hmmsearch tool was then applied to the *C. victoriae* venom gland transcriptome database of open reading frames.

All sequence alignments were performed with MAFFT version 7 using the L-INS-i method [75]. Signal peptide sequences were determined using the SignalP 4.1 server [76]. Mature peptide regions were predicted based on similarity to related conotoxin sequences.

### Availability of Supporting Data

Conotoxin prepropeptide sequences from this Transcriptome Shotgun Assembly project have been deposited at DDBJ/EMBL/GenBank [accession: GAIH00000000]. The version described in this paper is the first version, GAIH01000000. Raw sequencing data has been deposited in the NCBI sequence read archive [SRA accession: SRR833564].

### Ethics Statement

Specimens of *Conus victoriae* were collected specifically for research use, under a commercial fishing license of the Western Australian Specimen Shell Managed Fishery (license number 2577). Ethics approval was not required, in Australia, for taking samples from *Conus*.
